# Cell populations and muscle fiber morphology associated with acute and chronic muscle degeneration in lumbar spine pathology

**DOI:** 10.1002/jsp2.1087

**Published:** 2020-04-08

**Authors:** Bahar Shahidi, Michael C. Gibbons, Mary Esparza, Vinko Zlomislic, Richard Todd Allen, Steven R. Garfin, Samuel R. Ward

**Affiliations:** ^1^ Department of Orthopaedic Surgery University of California San Diego San Diego California USA; ^2^ Department of Bioengineering University of California San Diego San Diego California USA; ^3^ Department of Radiology University of California San Diego San Diego California USA

**Keywords:** fibroadipogenic (FAP) progenitor cell, low‐back pain, lumbar spine, muscle

## Abstract

Many chronic musculoskeletal conditions are associated with loss of muscle volume and quality, resulting in functional decline. While atrophy has long been implicated as the mechanism of muscle loss in these conditions, recent evidence has emerged demonstrating a degenerative phenotype of muscle loss consisting of disrupted muscle fiber membranes, infiltration of cells into muscle fibers, and as previously describer, possible replacement of muscle fibers by adipose tissue. Here, we use human lumbar spine pathology as a model system to provide a more comprehensive analysis of the morphological features of this mode of muscle loss between early and late stages of disease, including an analysis of the cell populations found in paraspinal muscle biopsies from humans with acute vs chronic lumbar spine pathology. Using longitudinal sections, we show that degeneration of muscle fibers is localized within a fiber (ie, focal), and is characterized by discontinuous or ragged membrane disruption, cellular infiltration, and apparently vacant space containing limited numbers of nuclei and hyper‐contractile cell debris. Samples from patients with acute and chronic pathology demonstrate similar magnitudes of muscle degeneration, however, larger proportions of PDGFRβ‐positive progenitor cells and leukocytes were observed in the acute group, with no differences in myogenic cells, macrophages, or T‐cells. By better understanding the cell population behaviors over the course of disease, therapies can be optimized to address the appropriate targets and timing of administration to minimize the functional consequences of muscle degeneration in lumbar spine pathology.

## Graphical abstract

Muscle degeneration is highly prevalent in acute and chronic lumbar spine pathology. Individuals with acute pathology demonstrate larger proportions of fibroadipogenic progenitor cells (FAPS) and leukocytes than those with chronic disease. These cells may contribute to the tissue compositional changes that lead to poor outcomes in lumbar spine pathology

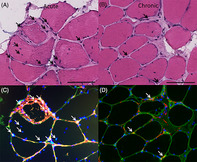

## 1 INTRODUCTION

Impaired muscle function is a hallmark of many musculoskeletal conditions that decrease quality of life for millions of patients annually.^1^, ^2^, ^3^ There are myriad causes of diminished muscle force production, ranging from simple loss of contractile protein volume, to sarcomere disorganization, to disruption in E‐C coupling.^4^, ^5^ In the first scenario, loss of functional contractile tissue can occur by either atrophy or degeneration. Therefore, these two terms are often used interchangeably in the clinical literature.^6^, ^7^ This is problematic because disparate and often competing biological mechanisms govern atrophy and degeneration respectively.^4^, ^5^ In turn, treatment modalities should be tailored to the underlying pathological mechanism, as failure to do so may lead to ineffective and possibly even detrimental effects of intervention.

Atrophy, which can be caused by mechanical or neuromuscular unloading, or unmet metabolic demand, is a well‐defined muscle‐intrinsic process mediated by the activation of the ubiquitin‐proteasome and autophagic pathways that actuate protein catabolism.^5^ On the single fiber level, atrophic fibers have smaller cytoplasmic volumes, but intact cellular machinery. By reloading the muscle it is possible to activate anabolic pathways and inhibit catabolic pathways, leading to muscle fiber hypertrophy, restoration of contractile tissue volume, and resulting in increased force production and improved function.^8^

In contrast, muscle degeneration is a broader term encompassing a wide array of muscle‐extrinsic physical and biochemical insults that lead to muscle fiber damage, which left unchecked, eventually leads to necrosis.^4^, ^9^ Classic examples are found in Duchenne muscular dystrophy and inflammatory myopathies, where muscle fiber membranes are disrupted by a specific protein deficiency and altered mechanical loading,^4^ or T‐cell mediated autoimmune attack,^10^ respectively. Although the term “degeneration” has been used to describe reductions in volume of functional contractile tissue in chronic musculoskeletal conditions (ie, lumbar spine pathology, rotator cuff disease), only recently, has the distinct physiological process of muscle degeneration been linked to these conditions, despite the lack of a clear primary etiology.^11^, ^12^ In a range of degenerative models, fibers display characteristics such as myophagocytosis and cellular infiltration, fiber splitting, and cytoplasmic disruptions (core, moth‐eaten, and targetoid fibers).^13^ These characteristics are also often paired with observations of increased presence of inflammatory markers.^12^, ^14^ Similar to atrophy, acute muscle degeneration does not necessarily lead to pathology, as cell death is typically followed by clearance of muscle fiber debris^15^ and satellite cell‐driven regeneration signified by a centralized myonucleus.^16^, ^17^ However, when regeneration is impaired or insufficient to replace degenerated fibers, or when the rate of degeneration outpaces regeneration, contractile tissue volume is reduced over time and often results in the accumulation of adipose tissue, or “fatty infiltration” in areas no longer occupied by muscle; a clinical observation that is commonly thought to be an indicator of poor muscle health or loss of whole muscle integrity.^17^ Importantly, with an atrophic mode of muscle loss, treatments aimed at blocking atrophy and/or promoting hypertrophy, such as resistance exercise, will likely improve outcomes. By contrast, with degenerative muscle loss, this same approach may not address the underlying pathology of muscle cell death. This idea is clinically supported by the lack of muscles' responsiveness to repair and rehabilitation in many patients with chronic musculoskeletal conditions.^18^, ^19^, ^20^, ^21^ It is unknown whether these two processes are related or occur within a specific disease time‐course.

In order to better understand, the degenerative process outside the context of specific genetic or immunogenic conditions with known causes of muscle degeneration (ie, muscular dystrophy, autoimmunity, or myotoxin), we focused on a clinical condition in which reduced muscle volume contributes to poor functional outcomes and decreased quality of life: lumbar spine pathology. Up to 85% of individuals experience low‐back pain within their lifetime,^22^ and low‐back pain is correlated with pathological changes of the bony and soft tissue structures that make up the spine.^23^ The lumbar spine muscles demonstrate decreased muscle cross‐sectional area (CSA) and evidence of fatty and fibrotic infiltration with advancing patient age and duration of disease,^24^, ^25^, ^26^ and also correlates with poor clinical outcomes.^27^, ^28^, ^29^ More recent evidence suggests that this population demonstrates a histotype of muscle degeneration^12^ similar to that found in rotator cuff tear,^11^ a condition which shares many other clinical and pathological features with lumbar spine pathology.^21^, ^29^ In both systems this newly characterized mode of degenerative muscle loss diverges from previous reports that suggest atrophy is the key mode of contractile tissue loss in chronic orthopedic disease, though the cellular and molecular mediators of this form of muscle degeneration, and the number of conditions that display this degenerative phenotype, remain unknown.

Here, we aimed to provide deeper insight into the cellular processes found in muscle degeneration and fatty and fibrotic infiltration by quantifying myogenic, adipogenic, and fibrogenic progenitor cell types as well as inflammatory cell types in in paraspinal muscle biopsies of individuals with acute and chronic lumbar spine pathology. We hypothesize that the morphology of degeneration is punctate and regional within a single fiber, and that this degeneration is associated with inflammatory macrophage activity. With these data, we expect to uncover new insights into the molecular mechanisms and time‐course of muscle fiber degeneration in lumbar spine pathology.

## 2 METHODS

### 2.1 Participants

This study was a prospective observational study. Intraoperative biopsies of the lumbar multifidus muscle were collected under appropriate Institutional Review Board approvals and all participants provided informed consent for this study (IRB 1611174). Biopsies were collected in patients undergoing posterior approach spinal decompression (discectomy, laminectomy, or laminoforaminotomy) or fusion surgical procedures for acute (symptom duration of <6 months, *N* = 10) or chronic (symptom duration >6 months, *N* = 22) degenerative lumbar spine pathology. Differences in patient demographic characteristics were statistically compared between acute and chronic groups using independent *t* tests for continuous variables (age, duration of symptoms), and chi squared tests for categorical or binary variables (gender, spinal levels). A *P* value of .05 was considered statistically significant. Muscle samples were pinned at in vivo length and flash frozen in liquid nitrogen cooled isopentane and stored at −80°C. Prior to sectioning, samples were embedded in optimal cutting temperature media, and histological sections were generated on a Leica cryostat at −20°C.

### 2.2 Histology

Axial and longitudinal biopsy sections cut on a cryostat (Leica CM3050 S) were stained with Hematoxylin and Eosin (H&E) and evaluated to describe the morphology and spatial pattern of degeneration within single fibers as well as the orientation of degenerating fibers relative to nonmuscle tissues and cells. A muscle fiber or region was considered to be degenerating if it demonstrated evidence of cytoplasmic disruption (ie, core, or moth‐eaten features), disruption or breakage of the muscle membrane, and/or cellular infiltration.^13^ Axial sections were used to identify the proportions of the overall biopsy that included degenerating regions, and to identify cells within those regions as previously described.^12^ Longitudinal sections were used to identify whether degeneration occurred throughout the entire length of the fiber or appeared to affect only specific regions throughout the length of the fiber. Fibers identified as degenerating in longitudinal sections were categorized based on the percentage of the fiber length that appeared to be degenerating (<25%, between 25% and 75%, and >75%), as well as whether those regions contained cytoplasmic disruptions, membrane disruptions, or cellular infiltration. To quantify specific cell types and structures within and around degenerating fibers, a sequential immunofluorescence/hematoxylin and eosin (IF/H&E) protocol was developed to allow identification of degenerating regions via H&E and cell type quantification via IF labeling in the same section, as detailed below. Each IF cell marker was identified on separate, but sequentially sectioned slides along with muscle membrane and nuclear counterstains. This approach was taken in place of absolute quantification from whole biopsy homogenate because it allows for the specific identification of degenerating regions.

### 2.3 Immunofluorescent staining

In order to optimize immunofluorescent staining to each antibody of interest, antibodies were fluorescently labeled with or without an antigen retrieval step. For antibodies requiring antigen retrieval (Pax7 and Myogenin), sections were fixed with 4% PFA followed by a 20% goat serum, 0.3% Triton X‐100 in PBS block. For antibodies not requiring antigen retrieval, sections were blocked in 1% bovine serum albumin in PBS. Inflammatory cell markers of macrophages (CD68, Abcam AB955, mouse, 1:50 dilution, secondary, Invitrogen AlexaFluor, IgG1 goat anti‐mouse 594, 1:500 dilution), leukocytes (CD45, Biolegend HI30, mouse, 1:500 dilution, secondary, Invitrogen AlexaFluor, IgG1 goat anti‐mouse 594, 1:500 dilution), and T‐cells (CD3, Biolegend SK7, mouse, 1:200 dilution, secondary, Invitrogen AlexaFluor, IgG1 goat anti‐mouse 594, 1:500 dilution) were used to label inflammatory cells on separate slides within and surrounding degenerating muscle fibers, which were counterstained with anti‐laminin (L9393, Sigma, rabbit, 1:1000 dilution, secondary, Invitrogen AlexaFluor, IgG goat anti‐rabbit 488 for all except PDGFRβ which was IgG chicken anti‐rabbit 488, 1:500 dilution) to visualize disrupted muscle fiber membranes and DAPI (VECTASHIELD Antifade Mounting Media with DAPI, H‐1200) to visualize nuclei. A similar strategy was used to quantify the localization of quiescent (Pax7, R&D Systems MAB1675, mouse, 1:100 dilution, secondary, Invitrogen AlexaFluor, IgG1 goat anti‐mouse 594, 1:500 dilution), activated (MyoD, Sigma M6190, mouse, 1:50 dilution, secondary, Invitrogen AlexaFluor, IgG2a goat anti‐mouse 594, 1:500 dilution), and committed (Myogenin, BD Pharmingen 556 358, mouse, 1:50 dilution, secondary, Invitrogen AlexaFluor, IgG1 goat anti‐mouse 594, 1:500 dilution) satellite cells as well as multipotent resident progenitors including pericytes and fibro/adipogenic‐progenitor (FAP) cells (PDGFRβ, R&D Systems AF385, goat, 1:100 dilution, secondary, Invitrogen AlexaFluor, IgG donkey anti‐goat 594, 1:500 dilution).

### 2.4 Cell quantification

Immediately after IF imaging using a microscope (Leica CTR6500, Buffalo Grove) fit with a fluorescent camera (Leica DFC365FX) using a ×10 objective, each slide was H&E stained. A single rater, who was blinded to the IF images, used the H&E images to identify degenerating regions of interest (ROIs) (defined by cellular infiltration of muscle fibers, membrane disruption, and/or cytoplasmic disruption (ie, core or targetoid fibers^11^, ^12^, ^13^). Then, using an overlay of the IF image, a separate blinded rater quantified the total number of mononuclear cells (including myonuclei) and the number of cells positive for the given IF marker in each ROI. For each IF/H&E slide, all identified regions of degeneration were quantified. A random sampling of at least 6, but no more than 10 regions of interest were quantified for each sample in both the nondegenerated and degenerated areas of muscle in acute and chronic biopsies. The total number of cells per region that were stained positively for each marker were averaged across regions and positive cell proportions were calculated as a percentage of total number of cells for each marker. All data is reported as mean (SD). Paired *t* tests were used to compare positive cell proportions between nondegenerating and degenerating regions within the same patient sample, and independent *t* tests were used to compare positive cell proportions from degenerating regions between patients with acute and chronic lumbar spine pathology. All analyses were performed using SPSS, version 26.0.0, IBM Corp. A *P* value of .05 was considered significant.

## 3 RESULTS

### 3.1 Participants

The average age of participants in the acute group was 62.6 (15.2) years old and 60.8 (15.8) years old in the chronic group (*P* = .78). There were no differences in the percentage of males vs females in the acute (70% vs 30%) and chronic (64% vs 36%) respectively (*P* = .80). The majority of the spinal muscle samples were obtained at the L4‐5 level (50%), followed by the L3‐4 level (28%), and finally the L5‐S1 level (15.6%), with no differences in the distribution of samples across levels between groups (*P* = .33). There was a significant difference in the duration of symptoms between those with acute (5.3 [1.3] months) vs chronic (67.9 [100.7] months) symptoms (*P* = .009).

### 3.2 Cell morphology

**FIGURE 1:**
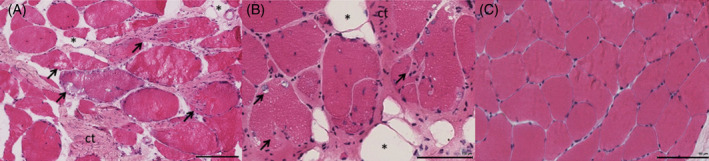
Axial Hematoxylin & Eosin (H&E) stains from the multifidus muscle in the spine in individuals with acute (A) and chronic (B) symptoms, in comparison with a representative image of a nondegenerating region (C). Black arrows indicate muscle fibers that demonstrate signs of degeneration; that is, cellular infiltration, membrane disruptions, and cytoplasmic disruptions. Large areas of connective tissue (ct) can be seen in‐between muscle fibers. Asterisks (*) denote adipocytes. Black scale bar represents 100 μm

**FIGURE 2:**
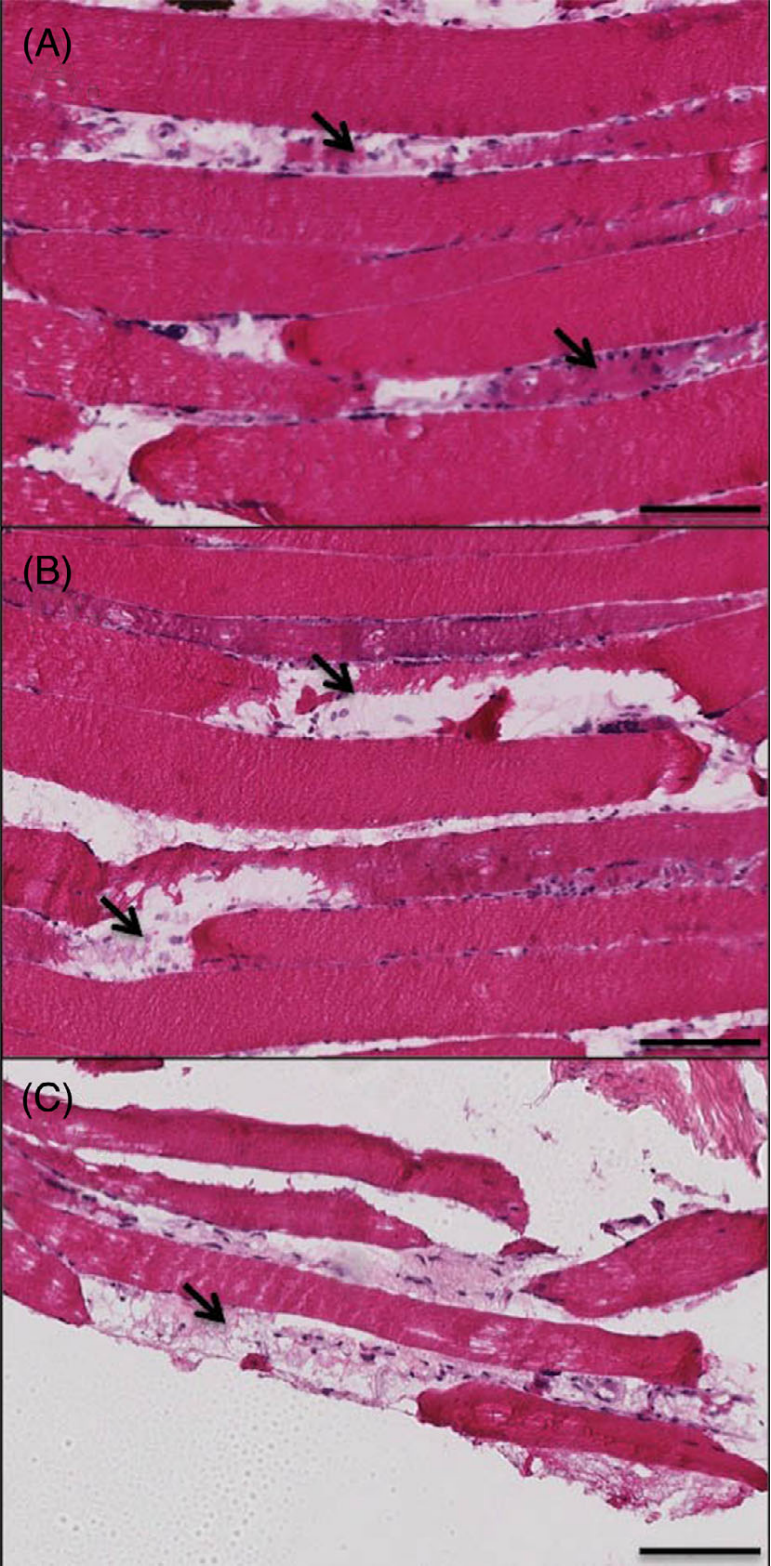
Representative Hematoxylin & Eosin stained sections demonstrating low (A), moderate (B), and severe, or end‐stage (C) muscle degeneration in the longitudinal plane of the multifidus muscle in the human spine in individuals with chronic symptoms. Areas of degeneration are signified by regions of hyper‐cellularity and adjacent empty space where the remaining portion of the muscle fiber would normally be (arrows). Images are magnified at ×20. Black scale bar represents 100 μm

In axial histological sections of human multifidus muscle from the spine, we noted a pattern of muscle degeneration characterized by distinct hypercellular regions within muscle fibers in which sarcolemmal integrity was compromised and sarcoplasmic volume was reduced (Figure 1A,B). When the amount of degeneration within a given sample was quantified from axial sections, there were no significant differences in the amount of muscle degeneration observed in samples with acute (53.3 [25.6]%, Figure 1A) vs chronic (52.9 [21.6]%, *P* = .96, Figure 1B) pathology. In the longitudinal plane, degeneration appeared to start at the muscle fiber membrane, where increased nuclei number and heterogeneous eosin staining combined with a jagged muscle fiber border represented the most common manifestation of the degenerative phenotype (Figure 2A, B). From this active region, the fibers demonstrated divergent phenotypes; in one direction, the fiber appeared normal, but in the other, the space occupied by the fiber did not show positive eosin staining, with smaller regions of ragged or hyper‐contracted cellular material consistent with muscle fiber necrosis, similar to the “ghost fibers” described by Webster et al^30^ (Figure 2C). An equal proportion of degenerating fibers appeared to have less than 25%, and between 25% and 75% of the visible muscle fiber length showing signs of degeneration (38.5% each). Only a quarter (25.6%) of the muscle fibers observed in the longitudinal plane demonstrated degeneration across more than 75% of their length, confirming the hypothesis that muscle degeneration is primarily a punctate and regional phenomenon. A large majority of degenerating regions were characterized by cytoplasmic disruption (82.1%), membrane disruption (74.4%), and cellular infiltration (82.1%).

### 3.3 Degenerative cell types

**FIGURE 3:**
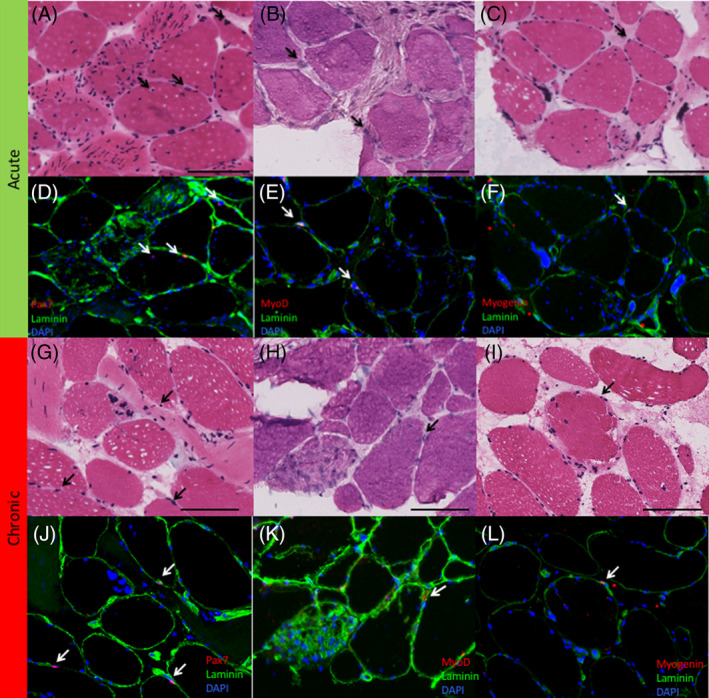
Immunohistochemistry stained sections of the multifidus muscle of the spine for (left column) Pax 7 positive cells, (middle column) MyoD positive cells, and (right column). Myogenin positive cells in regions of muscle degeneration in acute (top, A‐F) and chronic (bottom, G‐L) samples. Associated H&E stains for each section (Pax‐7—A, G; MyoD—B, H; Myogenin—C, I) are illustrated in the first and third rows. Black arrows indicate nuclei that stained positive for a cell marker in the associated IHC (white arrows) Black scale bar represents 100 μm

**FIGURE 4:**
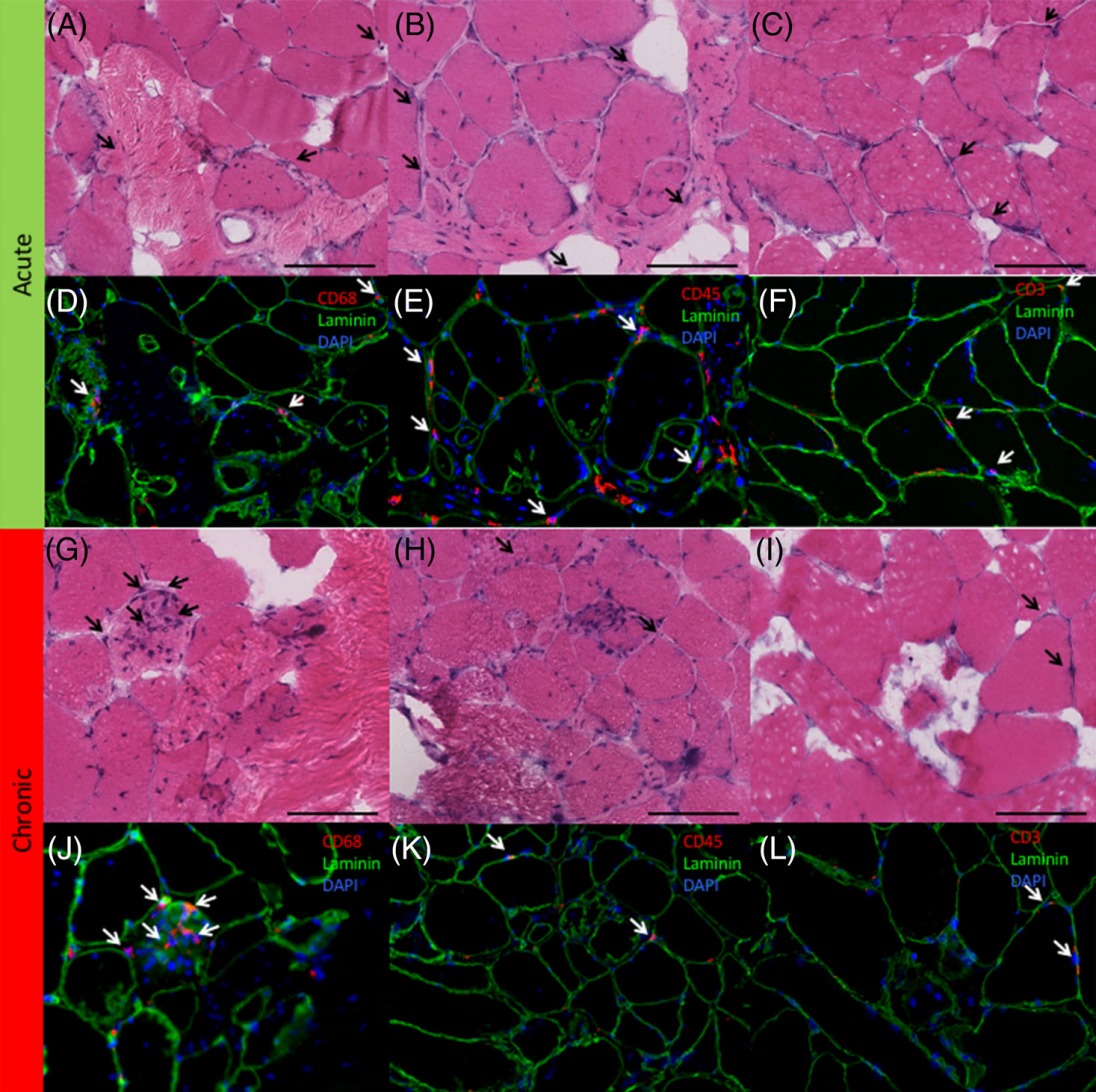
Immunohistochemistry sections of the lumbar multifidus muscle stained for (left column) CD68 positive macrophages, (middle column), CD45 positive leukocytes, and (right column) CD3 positive T cells in acute (top, A‐F), and chronic (bottom, G‐L) samples. Associated H&E stains for each section (CD68—A, G; CD45—B, H; CD3—C, I) are illustrated in the first and third rows. Black arrows indicate nuclei that stained positive for a cell marker in the associated IHC (white arrows) Black scale bar represents 100 μm

**FIGURE 5:**
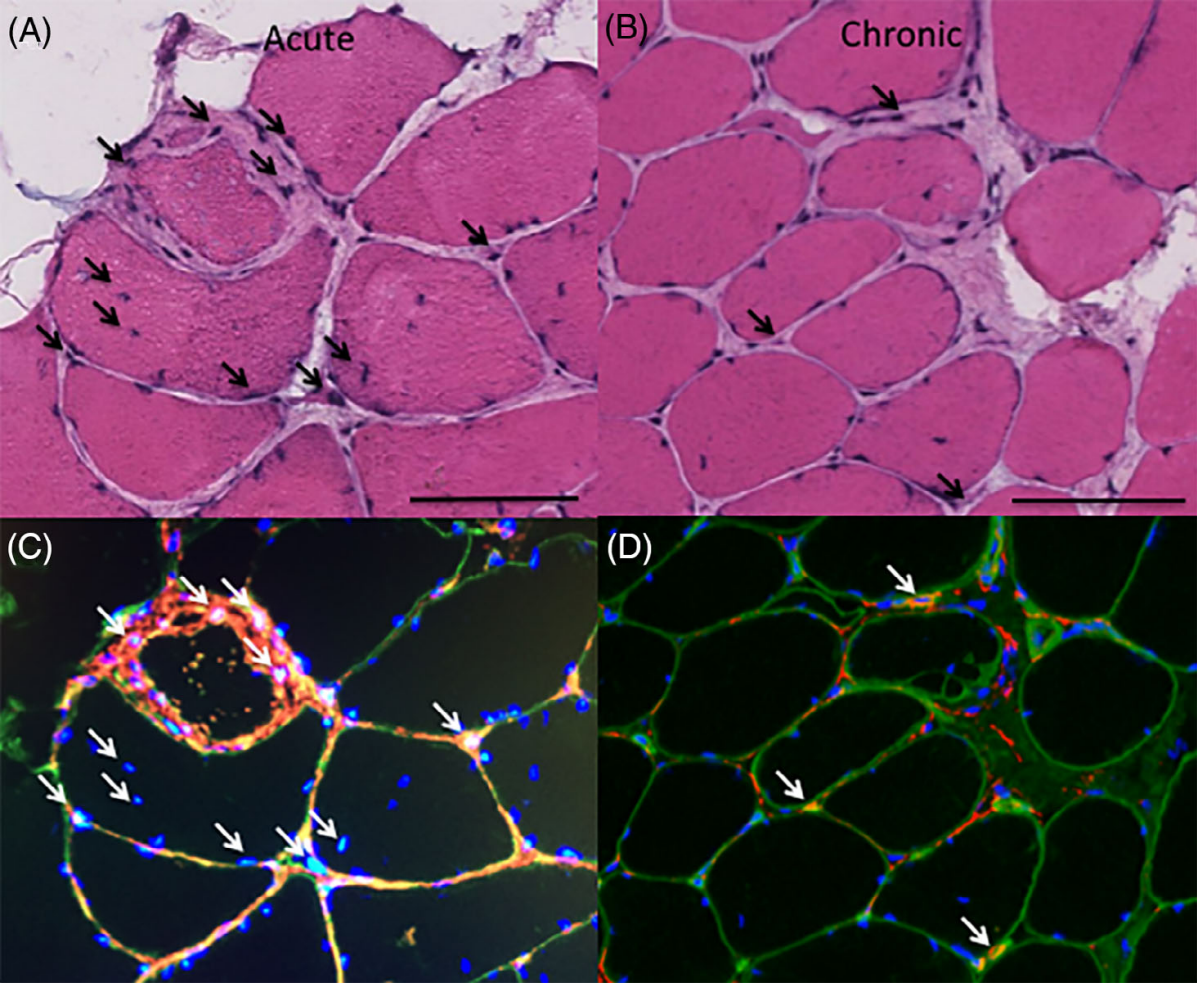
Immunohistochemistry sections of the lumbar multifidus muscle stained for PDGFRB positive fibro‐adipogenic progenitor cells along with their associated H&E sections for a representative acute (left—A, C) and chronic (right—B, D) sample. Gross projections of positive staining can be seen encompassing several nuclei within and around a degenerating muscle fiber, particularly in the acute sample. Black scale bar is 100 μm

**FIGURE 6:**
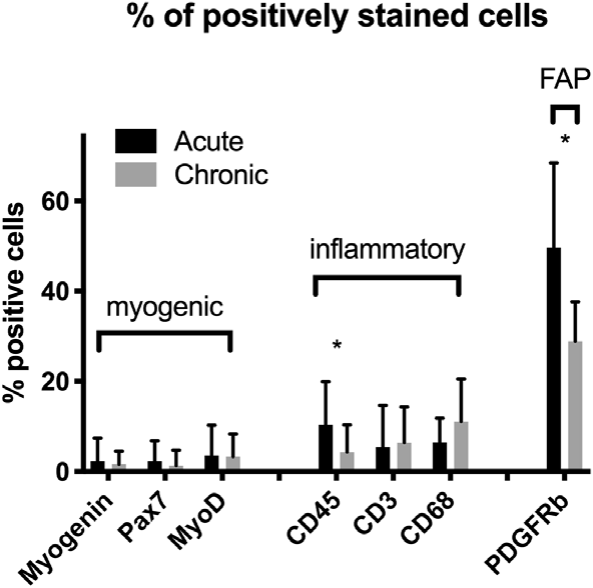
Bar plots illustrating positive cell proportions for each cell type in acute and chronic samples. Asterisks (*) indicate significant differences (*P* < .05) between measures in acute and chronic samples. Bars represent cell type mean percentages as a function of total number of nuclei/region means, and error bars represent SD

Myogenic cells, indicated by Pax7 (quiescent SCs) (Figure 3A,D), MyoD (activated SCs)(Figure 3B,E), and Myogenin (committed SCs) (Figure 3C,F), together made up less than 5% of the cells in degenerating regions on average, with a similar number and percentage found in acute and chronic samples (*P* > .26). Inflammatory cells comprised between 6% and 16% of cells in degenerating regions, with an average of 10.2 (8.9)% of cells positive for CD68 (macrophages) (Figure 4A,D), 7.8 (8.6)% of cells positive for CD45 (pan‐leukocyte) (Figure 4B,E) and 5.7 (8.8)% of cells positive for CD3 (pan T‐cell) Figure 4C,F). There was a significantly higher percentage of cells positive for CD45 in the acute (10.4 [9.6] %) vs chronic (4.4 [5.9]% samples, *P* = .03), with no significant differences between groups for the other inflammatory cells (*P* > .36). The most prolific cell marker observed was for the multipotent stem cell marker PDGFRβ. In the setting of histologically normal fibers, these cells typically have few projections, however, in degenerating regions, PDGFRβ‐positive projections were found surrounding, and in some cases even penetrating into, degenerating fibers (Figure 5A,C). There was a significantly larger percentage of PDGFRβ positive nuclei in acute (49.7 [18.7]%) (Figure 5A,C) vs chronic (28.9 [8.7]%) (Figure 5B,D) samples (*P* = .04). Proportions of cell types in acute and chronic samples are illustrated in Figure 6.

### 3.4 Regions of nondegenerated muscle

**FIGURE 7:**
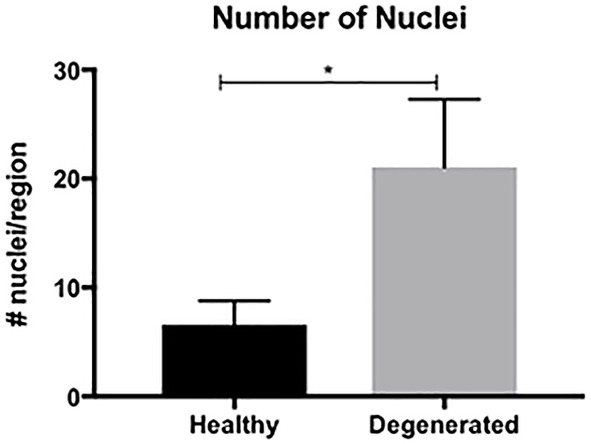
Bar plot illustrating the average number of nuclei quantified within nondegenerating (black bar) and degenerating (gray bar) regions. Bars represent mean number of nuclei quantified/region, and error bars represent SD

**FIGURE 8:**
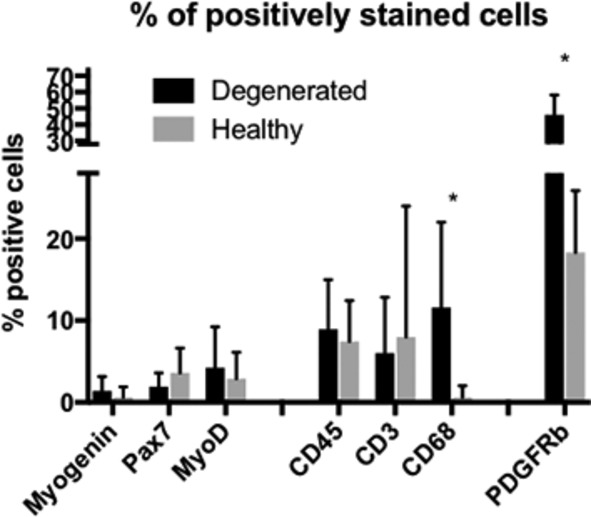
Bar plots illustrating positive cell proportions for each cell type in nondegenerating (gray bars) and degenerating (black bars) regions. Asterisks (*) indicate significant differences (*P* < .05) between measures in nondegenerating and degenerating regions. Bars represent cell type mean percentages as a function of total number of nuclei/region means, and error bars represent SD

Based on our observation that muscle degeneration seems to be a regional phenomenon and was equally prevalent in both acute and chronic individuals, we were also interested in how the cell type proportions differed between nondegenerating and degenerating regions of muscle within the same biopsy sample across both groups. In the absence of true control tissue, this comparison could provide information on relative proportions of cell types that are specifically involved in the degeneration, as opposed to cell types that are present in morphologically intact areas of muscle. In order to further investigate this phenomenon, we identified regions of muscle within each quantified sample that did not have any morphological signs of cellular infiltration, degeneration, fibrosis, or fatty infiltration (Figure 1C). Quantifications of cell types were performed in a similar manner for comparison between nondegenerating and degenerating regions. In this subanalysis, we found that there were a larger total number of nuclei present in the degenerating regions as compared to the nondegenerating regions (Figure 7) (*P* < .001). Despite greater nuclear numbers, the proportions of positive nuclei for all cell types were not different between nondegenerating and degenerating regions (*P* > .27) with the exception of CD68 positive macrophages (*P* = .045) and PDGFRβ+ FAP cells (*P* = .008), where degenerating regions had higher proportions of both (Figure 8).

## 4 DISCUSSION

The key findings in this paper are that degenerating regions of muscle are highly prevalent in both acute and chronic stages of lumbar spine pathology, and appear to be punctate and regional within a single muscle fiber. Contrary to our hypothesis, we found that the most common cell type observed within these degenerating regions were positive for pericyte or fibro‐adipogenic‐progenitor cells as opposed to inflammatory macrophages. These FAP cells were particularly apparent in the acute phase, along with a slightly larger proportion of leukocytes when compared to muscle from patients with chronic disease. Interestingly, other inflammatory cells (macrophages and T‐cells) and myogenic cells did not differ between acute and chronic patients. These data are the first to describe the regional nature of muscle degeneration in musculoskeletal disease, and to quantify distributions of cell populations in localized areas of degenerating muscle across different stages of lumbar spine disease in humans.

The large proportion of PDGFRβ‐positive cells in degenerating regions suggests that this cell type may be a key player contributing to the tissue compositional changes observed with degeneration. While the precise cell population demarcated by PDGFRβ positivity is still debated,^31^, ^32^ in healthy muscle these cells are reported to have a supportive role of myogenesis via phagocytosis of debris from degenerating fibers,^33^ secretion of promyogenic extracellular matrix and growth factors,^32^, ^34^, ^35^ and to a lesser extent, direct fusion with regenerating fibers. However, when the coordination of inflammatory cues is disrupted, as appears to be the case in lumbar spine pathology and other chronic orthopedic conditions, these cells may take on fibroblastic or adipogenic phenotypes.^32^, ^34^ Indeed, this cell population has been implicated in the terminal fatty and fibrotic composition of muscles in many diseases.^11^, ^31^, ^32^ Additionally, the influence of inflammatory cells on FAP function and regulation in muscle, specifically through the induction of FAP cell apoptosis, has been observed in muscle injury models^36^ and even nonmusculoskeletal conditions such as chronic kidney disease.^37^ Here, PDGFRβ‐positive cells appear to penetrate into fibers with otherwise normal‐appearing cytosol and intact membranes distal to the point of disruption, which suggests a more active role for these cells in the initiation and propagation of fiber degeneration in the early phases of disease.

The potential disruption of the normal role of PDGFRβ in supporting myogenesis is also supported by the observation that a proportional increase in myogenic cells was not observed in the acute samples despite the higher percentages of PDGFRβ‐positive cells. In otherwise healthy muscle, fiber death is followed by mobilization and expansion of satellite cells and other progenitor cells (most notably pericytes and fibro/adipogenic progenitors) that together mediate muscle regeneration.^9^, ^16^, ^17^, ^33^, ^35^, ^38^ Here, the fraction of satellite cells relative to total nuclei was not different between acute and chronic regions, and was consistent with previously reported satellite cell fractions in healthy muscle.^39^ This may provide insight into the irreversibility of muscle degeneration in lumbar spine pathology and similar orthopedic conditions—even in the early stages of symptom manifestation.

One interesting observation was the relatively low proportion of inflammatory cells in degenerating regions, even in the acute stage of pathology. Although there were significantly higher levels of CD45‐positive leukocytes present in the acute samples, the overall proportions of inflammatory cells were surprisingly low given the profound levels of degeneration observed throughout the muscle tissue. We have previously demonstrated that inflammatory cells are present at high levels in muscle biopsies of individuals with chronic lumbar spine pathology,^12^ however, in these observations the inflammatory cells present were often not located within the regions of cellular degeneration, but instead in the interstitial space between muscle fascicles, or in areas of muscle that did not demonstrate a degenerative phenotype. This observation may suggest that inflammatory cells are regionally dysregulated in degenerating muscle, or that the activation of inflammatory cells occurs prior to the manifestation of the degenerative phenotype. Because it was not possible to track active muscle degeneration over time within a single sample, it was not possible to distinguish between these two hypotheses. This observation may provide important context for future studies in animal models with the methodological tools to investigate the time‐course of these degenerative phenomena with more granularity.

A primary limitation of work was our inability to resolve these quantifications in relation to control spinal tissue from normal healthy individuals. The ability to obtain spinal muscle biopsies of similar depth and standardized location accuracy in healthy individuals is difficult for ethical and methodological reasons and remains an important gap in the literature. Similarly, given the high prevalence of low‐back pain in the general population (65%‐85%), using cadaveric tissue as a comparison is also suboptimal because very few specimens can be considered to be true controls. Importantly, low‐back pain or associated pathology is often undocumented prior to death in these specimens. Although biopsies from other more accessible anatomical locations may be an alternative strategy, the paraspinal muscle has unique properties that makes comparisons to other muscles suboptimal.^40^, ^41^ However, muscle biopsies from other muscles may still be informative in the absence of normal paraspinal tissue. The only other study that has quantified FAPs in pathological human muscle biopsies was performed in individuals who had an anterior cruciate ligament (ACL) injury. In the aforementioned study, FAP quantifications from biopsies of the vastus lateralis muscle were compared between injured and uninjured extremities and reported that the FAP density was roughly double in the injured samples as compared to the uninjured samples.^42^ In the absence of available control tissue in the spine, the comparison of morphologically degenerating regions to morphologically intact regions of muscle may provide some initial insight into the degenerative process, despite the fact that these samples as a whole cannot be considered “normal.”

Another important limitation is that, due to heterogeneity of tissue health throughout the sample (ie, on average 50% of the muscle was degenerated, and 50% demonstrated no visible evidence of pathology), whole tissue quantification of the markers of interest was not possible through methods such as ELISAs or Western Blots without losing the ability to distinguish regional tissue health.

Beyond our limited ability to reference differences in cellular characteristics to healthy control tissue, we are also limited by the heterogeneity across patients in terms of disease etiology. Specifically, low‐back pain is a complex condition that may involve a variety of sources of symptom‐inducing pathologies, from neurologic compromise to arthritis. Although the majority of the patients in this sample were undergoing surgery for a combination of degenerative disc disease, facet joint arthritis, and some form of central or foraminal stenosis, the extent to which any individual pathology differentially contributes to muscle atrophy and/or degeneration was beyond the scope of this study and may be an interesting future direction of research in this area.

## 5 CONCLUSIONS

Muscle volume loss and fatty infiltration are common features in chronic degenerative musculoskeletal conditions such as lumbar spine pathology. However, the mechanisms underlying the loss of functional contractile tissue in these conditions are more complex than simple atrophy. Here, we demonstrate that muscle degeneration, characterized by punctate regions of muscle fiber necrosis displaying both hyper‐ and hypo‐cellular regions in the same fiber, is a common feature of spine muscle pathology. Innate and adaptive immune cells and multipotent resident progenitors are more prevalent than myogenic satellite cells in degenerative regions, which support the hypothesis that limited regeneration combined with inflammatory signals and immune cell‐derived products, influence muscle degeneration in patients with chronic orthopedic disease. Furthermore, these changes occur early in the stages of disease and do not seem to continue progressing after initiation. By improving our understanding of the degenerative mechanisms that drive this particular form of muscle loss, we will facilitate the improvement of strategies aimed at preventing degeneration early in the disease process and stimulating regeneration of muscle tissue in these patients.

## CONFLICT OF INTEREST

The authors have no conflicts to declare.

## AUTHOR CONTRIBUTIONS

Bahar Shahidi designed the experiment, collected and analyzed data and wrote the manuscript. Michael C. Gibbons analyzed the data and wrote the manuscript. Mary Esparza analyzed data and edited the manuscript. Vinko Zlomislic, R. Todd Allen, and Steven R. Garfin collected the data and edited the manuscript. Samuel R Ward was involved in the experimental design, interpretation of data, and edited the manuscript. All authors have read and approved the final manuscript.
